# Water and lipid content of breast tissue measured by six-wavelength time-domain diffuse optical spectroscopy

**DOI:** 10.1117/1.JBO.27.10.105002

**Published:** 2022-10-13

**Authors:** Hiroko Wada, Nobuko Yoshizawa, Etsuko Ohmae, Yukio Ueda, Kenji Yoshimoto, Tetsuya Mimura, Hatsuko Nasu, Yuko Asano, Hiroyuki Ogura, Harumi Sakahara, Satoshi Goshima

**Affiliations:** a Hamamatsu Photonics K.K., Central Research Laboratory, Hamamatsu, Japan; b Hamamatsu University School of Medicine, Department of Radiology, Hamamatsu, Japan; c Hamamatsu University School of Medicine, Department of Breast Surgery, Hamamatsu, Japan; d Higashiomicity Gamo Medical Center, PET Center, Higashiomishi, Japan

**Keywords:** diffuse optical spectroscopy, time-resolved spectroscopy, dense breast, water, lipids

## Abstract

**Significance:**

The water and lipid content of normal breast tissue showed mammary gland characteristics with less influence from the chest wall using six-wavelength time-domain diffuse optical spectroscopy (TD-DOS) in a reflectance geometry.

**Aim:**

To determine the depth sensitivity of a six-wavelength TD-DOS system and evaluate whether the optical parameters in normal breast tissue can distinguish dense breasts from non-dense breasts.

**Approach:**

Measurements were performed in normal breast tissue of 37 breast cancer patients. We employed a six-wavelength TD-DOS system to measure the water and lipid content in addition to the hemoglobin concentration. The breast density in mammography and optical parameters were then compared.

**Results:**

The depth sensitivity of the system for water and lipid content was estimated to be ∼15  mm. Our findings suggest that the influence of the chest wall on the water content is weaker than that on the total hemoglobin concentration. In data with evaluation conditions, the water content was significantly higher (p<0.001) and the lipid content was significantly lower (p<0.001) in dense breast tissue. The water and lipid content exhibited a high sensitivity and specificity to distinguish dense from non-dense breasts in receiver-operating-characteristic curve analysis.

**Conclusions:**

With less influence from the chest wall, the water and lipid content of normal breast tissue measured by a reflectance six-wavelength TD-DOS system, together with ultrasonography, can be applied to distinguish dense from non-dense breasts.

## 1 Introduction

Diffuse optical spectroscopy (DOS) is an emerging modality being researched for the examination of breast tissue and diagnosis of breast cancer.^1^^–^^4^ DOS enables quantitative measurements of oxygenated and deoxygenated hemoglobin concentrations, which are directly related to tumor angiogenesis.^5^

Measurements of water and lipid content of breast cancer can provide important information about the characteristics of a tumor. Chung et al.^6^ reported that high water content in a tumor indicates edema and increased cellularity. Cerussi et al. reported that cancerous breast lesions contain more water but fewer lipids than normal breast tissue.^7^ For quantitative measurements of water and lipid content in addition to hemoglobin concentration measurement using a conventional device (TRS-20SH, Hamamatsu Photonics K.K., Hamamatsu, Japan), we previously developed a six-wavelength time-domain DOS (TD-DOS) system with three additional wavelengths to measure absorption specific to water and lipids (TRS-21-6W, Hamamatsu Photonics K.K.).^8^ We previously reported the possibility of combining TD-DOS with ultrasonography (US) by performing reflectance measurements with a three-wavelength TD-DOS system capable of determining the tissue concentration of oxyhemoglobin (O_2_Hb), deoxyhemoglobin (HHb), total hemoglobin (tHb), and tissue oxygen saturation.^9^^–^^12^ We examined the relationships between these parameters and structural information for breast tissue. As a result, we found that tHb can be used as a biomarker to evaluate breast cancer, although the effect of the chest wall must be considered.^9^^,^^10^ Calculations in the TD-DOS measurements were conducted under the assumption of homogeneity in the measurement area; however, normal breast tissue actually has a layered structure comprising skin, lipids, mammary gland, and chest wall. When evaluating breast cancer using the device, a mass would be added to this layered structure. In measurements obtained by the three-wavelength TD-DOS system, there were some cases in which the deepest chest wall layer displayed a strong influence on the results.^9^^,^^10^ To address these concerns, we used US in our study and reported the relationship between the measurement data and the breast structure based on a comparison of US images and optical data. Because six-wavelength TD-DOS measurements were acquired in a reflectance geometry similar to a three-wavelength system, it was necessary to study the effect of each tissue layer on the optical parameters. To aid in evaluating optical data in breast cancer, it is important to carefully study structural information for normal breast tissue, which has a simpler layered structure than cancerous breast tissue. It is also necessary to study the depth sensitivity of water and lipid content for evaluating normal breast and then breast cancer.

It is valuable to identify dense breasts using near-infrared measurement, which avoids radiation exposure. Breast cancer risk varies by race and country; however, there is a common issue for women with dense breasts. In mammography (MMG) results, a higher breast density correlates with an increased risk of breast cancer.^13^^–^^16^ Some studies have shown a direct correlation between the breast imaging reporting and data system (BI-RADS) classification for MMG breast density and water content.^17^ Altoe et al.^18^ reported that the water percentage of normal breast tissue correlated significantly with breast density. Asian women tend to have denser breasts than American women.^19^ There is a higher risk for overlooking cancer in mammograms of dense breasts because the breast lesion is covered by surrounding fibroglandular tissue. Prior information on patients with dense breasts might prompt the use of additional modalities such as magnetic resonance imaging (MRI) and US, depending on the patient’s family history, genetic testing, and other risks of breast cancer. Although no relationship has been found between a mortality reduction in breast cancer and the use of US and/or MRI in addition to MMG, US and MRI contribute to early detection of breast cancer.^20^^–^^24^ Informing patients about their dense breasts is also considered to be an effective way to promote breast cancer awareness, which includes self-examination and voluntary breast screening.

According to a previous study, there was a correlation between some physical parameters obtained by near-infrared-transmittance measurements and breast density in MMG.^25^ In this study, we compared optical data measured in the reflectance geometry with MMG breast density to determine whether optical data can distinguish dense and non-dense breasts.

## 2 Materials and Methods

### 2.1 Subjects

This study included 48 patients who were diagnosed with breast cancer from September 2017 to November 2018. Patients who received neoadjuvant chemotherapy (n=8) or hormone therapy (n=3) were excluded, leaving 37 patients. All were Asian women with an age range of 25–88 years, median age of 65 years, and median body mass index of 22.8 (range: 16.4–32.4). Nine patients were premenopausal and 28 were postmenopausal. In this study, only data for normal-side breasts were evaluated. The study protocol was approved by the Ethical Review Committee of the Hamamatsu University School of Medicine, and written informed consent was obtained from all patients.

### 2.2 TD-DOS and US Measurements

The optical properties of breast tissue were measured using a six-wavelength TD-DOS system (TRS-21-6W, Hamamatsu Photonics K.K., Hamamatsu, Japan). The system was developed based on a three-wavelength TD-DOS system (TRS-20SH, Hamamatsu Photonics K.K.) used in our previous studies.^9^^–^^12^

The TRS-21-6W system uses the time-correlated single-photon counting (TCSPC) method to acquire the time response profile of pulsed laser light penetrating the target tissue. The system includes two light source units, each containing three laser diodes (762, 802, and 838 nm for the first unit; 908, 936, and 976 nm for the second unit) that emit a light pulse with a full width at half maximum of ~100 to ~200 ps at a repetition rate of 5 MHz (custom-designed, Hamamatsu Photonics K.K.). The photodetector units consist of two photomultiplier tubes (GaAs and InGaAs, Hamamatsu Photonics K.K.), constant fraction discriminators, time-to-amplitude converters, analog-to-digital converters, and histogram memories. The six pulsed lasers are integrated into one source fiber with a diameter of 1 mm and a numerical aperture of 0.29 (Sumita Optical Glass, Inc., Saitama, Japan). The irradiated light from the source fiber is collected by a detection fiber bundle with a diameter of 3 mm and a numerical aperture of 0.29 (Sumita Optical Glass, Inc.). The specifications and analysis method of the improved system are described in detail in Ref. 8. The system uses the TCSPC method to acquire the temporal response profiles of tissue from optical pulse inputs, which enable quantitative analyses of light absorption and scattering in tissue based on photon diffusion theory.^26^

The water and lipid content and the concentrations of $O_{2}\mathrm{Hb}$ and HHb are calculated from the absorption coefficients for the six wavelengths. The concentration of tHb is the sum of the $O_{2}\mathrm{Hb}$ and HHb concentrations. As described in our previous study,^8^ the water and lipid content are the volume fractions of water and lipids measured by the TRS-21-6W system. Notably, these are relative values compared with those of pure solutions and not the actual composition ratios of the tissue.^27^ The measurement accuracy of the absorption and scattering coefficients and the adequacy of the water and lipid content were evaluated in Ref. 8.

**Fig. 1:**
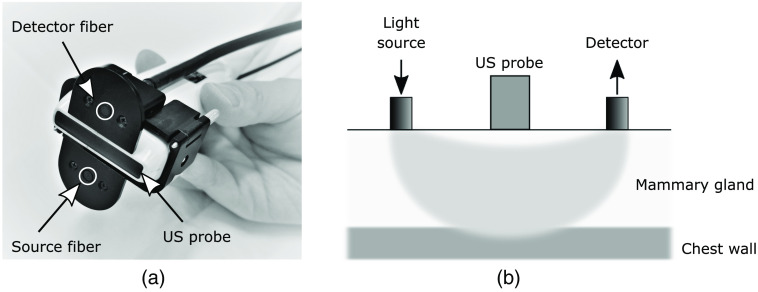
(a) Optical probe attached to an ultrasound (US) probe. (b) Schematic illustration of the diffusion of light propagating in the breast and positioning of the optical fibers and US probe.

Breast measurements were carried out in the reflectance geometry with a source–detector separation of 3 cm. The measurements were performed in supine position and performed simultaneously with the TRS-21-6W system and an US system (EUB-7500; Hitachi Medical Corporation, Tokyo, Japan) using a custom spectroscopic probe in which the resulting optical path is set orthogonal to the US image. The probe was attached to a linear probe (EUP-L65, Hitachi Medical Corporation), as shown in Fig. 1(a), and lightly pressed on the breast to measure the absorption and reduced scattering coefficients at six wavelengths while simultaneously obtaining US images. The light from the source fiber is scattered and absorbed in the medium before it reaches the detector. The photon path between the source and the detector fiber is considered to be banana-shaped [Fig. 1(b)].^28^

### 2.3 Mammography

MMG results obtained at our current or former hospital on the nearest date to the optical measurement were categorized into four types based on BI-RADS 5th ed.: (a) the breasts are almost entirely fatty; (b) there are scattered areas of fibroglandular density; (c) the breasts are heterogeneously dense, which may obscure small masses; and (d) the breasts are extremely dense.

### 2.4 Measurement and Evaluation Procedures

Measurements were performed at several locations on the normal-side breast. Our evaluation was first conducted on data that had a two-layer structure of lipids and the chest wall but no mammary gland in the subclavian area or in breast area away from a nipple, as confirmed by simultaneous US images. Data were obtained for 38 locations from 25 patients, with a maximum of four locations for each patient.

To evaluate normal breast data, data for 133 locations were included. Measurements were performed on normal breast tissue at a maximum of six locations for each patient. We excluded data measured directly on the nipple.

**Fig. 2:**
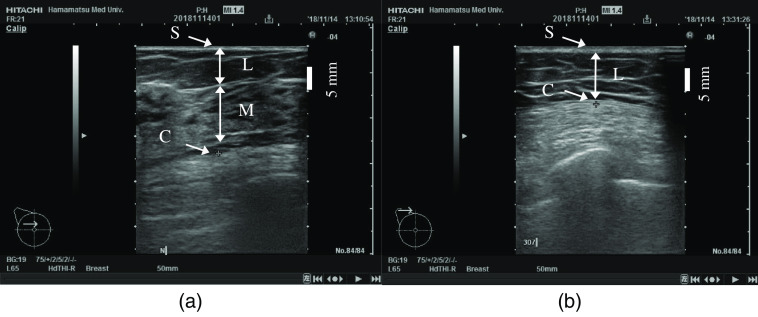
(a) US image of normal breast tissue and (b) the subclavian area with a two-layer structure of lipids and the chest wall. The images show the skin (S), lipid layer (L), mammary gland (M), and chest wall (C).

Figure 2 shows US images of normal breast tissue and of tissue in the subclavian area with a two-layer structure of lipids and the chest wall. The skin-to-mammary gland distance and the skin-to-chest wall distance were measured at the center of US images. To measure the skin-to-chest-wall distance, the chest wall was defined as the surface of the major pectoral muscle or anterior serratus muscle.

A previous study^9^ showed that the tHb value increased as the skin-to-chest wall distance decreased, indicating that the chest wall has a strong influence on tHb measurements. To study the influence of the chest wall on water and lipid content, we examined the relationship between water and lipid content and the skin-to-chest wall distance for the two-layer structure of a lipid layer and chest wall using the sigmoid curve-fitting method. We also compared the data with tHb measurements for the same structure.

The following regression equation was selected for the sigmoid curve based on previous studies:^29^^,^^30^

$$f=y_{0}+a/(1+\exp(-(x-x_{0})/b)),$$ (1)

where x is the skin-to-chest wall distance (mm); f is the tHb concentration (μM), water content (%), or lipid content (%); and $y_{0}$, $x_{0}$, a, and b are constants determined for tHb, water content, and lipid content, respectively. The $y_{0}$ value corresponds to the fitted curve’s plateau in tHb or water content, which should represent the corresponding content of the superficial lipid layer in a two-layered structure. The value of $y_{0}+a$ indicates the fitted curve’s plateau in lipid content, which should represent the corresponding content of the superficial lipid layer in two-layered structure.

Next, we plotted the tHb concentration, water content, and lipid content of normal breast tissue against the skin-to-chest wall distance and skin-to-mammary gland distance. The tHb concentration result was used as a reference. We also applied the sigmoid curve-fitting method to the tHb concentration, water content, and lipid content.

Two expert radiologists assigned BI-RADS MMG density categories for all 37 patients. When images were classified into different categories by each radiologist, the final classification was determined after discussion.

We compared the tHb concentration, water content, and lipid content in normal breast tissue for each MMG category. To assess the potential to distinguish dense from non-dense breasts based on optical data, the tHb concentration, water content, and lipid content for all 133 data points were shown separately with skin-to-chest wall distance in dense and non-dense breasts. Boxplots were used to display the water and lipid content for locations at which the skin-to-chest wall distance was large enough to avoid the effect of the chest wall and the skin-to-mammary gland distance was small enough to represent the mammary gland. The optical data were then analyzed using receiver-operating-characteristic (ROC) curve analysis to determine their potential to distinguish dense from non-dense breasts.

### 2.5 Statistical Analysis

Statistical analyses were performed using Microsoft Excel 2016 (Microsoft Corporation, Redmond, Washington), StatFlex version 6.0 (Artech Co., Ltd, Osaka, Japan), and SigmaPlot version 14.0 (Systat Software, Inc., San Jose, California). A sigmoid curve (1) was applied for curve fitting of the tHb, water content, and lipid content in the two-layer structure and in normal breast tissue.

The tHb concentration and water and lipid content in normal breast tissue in each MMG category were compared using nonparametric Mann–Whitney U tests with Bonferroni correction. The skin-to-mammary gland distances of dense and non-dense breasts were compared using nonparametric Mann–Whitney U tests. The water and lipid content of dense and non-dense breasts were compared using nonparametric Mann–Whitney U tests. Differences with p<0.05 were considered significant.

To identify an optimal threshold for discriminating dense from non-dense breasts, ROC curve analysis of optical data was performed by incrementally increasing the cutoff values. The area under the curve (AUC), sensitivity, specificity, positive predictive value (PPV), negative predictive value (NPV), and accuracy were analyzed.

## 3 Results

### 3.1 tHb Concentration, Water Content, and Lipid Content in the Two-Layer Structure of Lipids and the Chest Wall

**Fig. 3:**
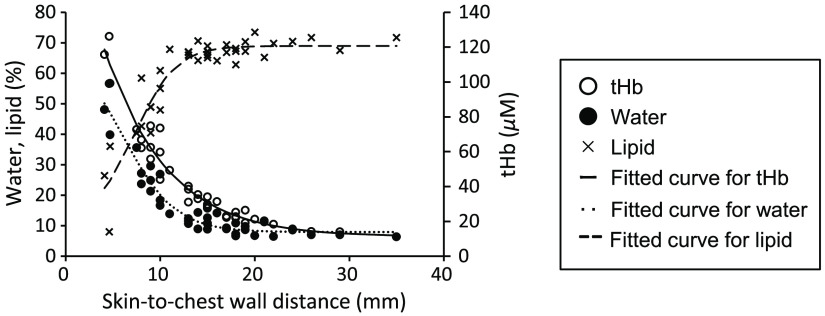
tHb concentration, water content, and lipid content in the two-layer structure versus skin-to-chest wall distance. Experimental data (n=38) and fitted curves are shown for tHb concentration (R=0.978), water content (R=0.965), and lipid content (R=0.931).

The tHb concentration, water content, and lipid content in the two-layer structure comprising a lipid layer and the chest wall are shown in Fig. 3. The tHb concentration increased as the skin-to-chest wall distance decreased from ∼20  mm. The water content increased and the lipid content decreased as the distance decreased from ∼15  mm. The value of $y_{0}$ in the sigmoid curve (1) was 11.1 for tHb concentration, 7.9 for water content, and the value of $y_{0}+a$ was 69 for lipid.

### 3.2 tHb Concentration, Water Content, and Lipid Content in Normal Breast Tissue

**Fig. 4:**
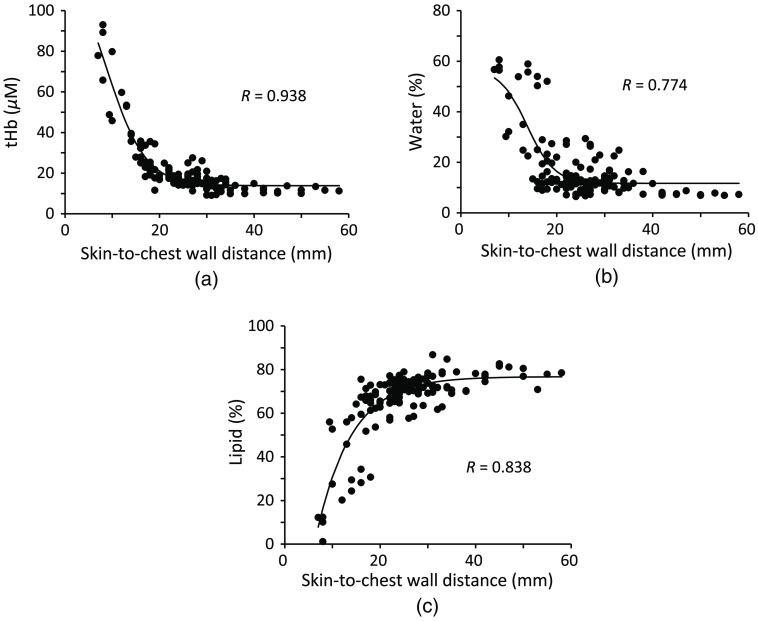
tHb concentration, water content, and lipid content of normal breast tissue versus skin-to-chest wall distance. Experimental data (n=133) and fitted curves are shown for (a) tHb concentration (R=0.938), (b) water content (R=0.774), and (c) lipid content (R=0.838).

The tHb concentration, water content, and lipid content in normal breast tissue for all 133 data in 37 patients are shown in Fig. 4. In the figure, the relationship between each parameter and the skin-to-chest wall distance is displayed. Both tHb concentration and water content increased as the skin-to-chest wall distance decreased, whereas the lipid content decreased as the skin-to-chest wall distance decreased. The multiple correlation coefficients (R values) for the fitted curves were 0.938 for tHb, 0.774 for water content, and 0.838 for lipid content. The coefficients of variation were calculated to compare the dispersion of tHb concentration and water content in normal breasts for data with a skin-to-chest wall distance of 20 mm or more, assuming that the chest wall has little effect on each parameter. The coefficients of variation were 24.3% for tHb concentration and 41.4% for the water content.

**Fig. 5:**
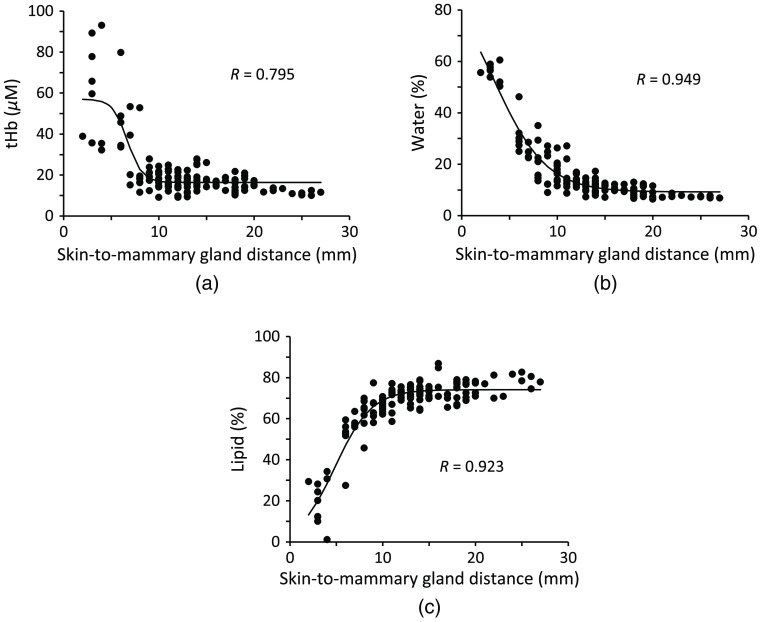
tHb concentration, water content, and lipid content of normal breast tissue versus skin-to-mammary gland distance. Experimental data (n=133) and fitted curves are shown for (a) tHb concentration (R=0.795), (b) water content (R=0.949), and (c) lipid content (R=0.923).

Figure 5 shows the relationship between tHb concentration, water content, and lipid content and the skin-to-mammary gland distance in normal breast tissue for all 133 data from 37 patients. Both the tHb and water content increased as the skin-to-mammary gland distance decreased. Lipid content decreased as the skin-to-mammary gland distance decreased. For skin-to-mammary gland distances <10  mm, the dispersion of tHb values was large, whereas the values of water and lipid content lie near the fitted curves, showing a distribution similar to that of the two-layer structure of a lipid layer and chest wall. The values of R for the fitted curves were 0.795 for tHb, 0.949 for water content, and 0.923 for lipid content.

### 3.3 tHb Concentration, Water Content, and Lipid Content in Normal Breast Tissue Based on Breast Density

The breast density in MMG was classified as “almost entirely fat” (category a) in three patients, having “scattered fibroglandular densities” (category b) in 20 patients, “heterogeneously dense” (category c) in 11 patients, and “extremely dense” (category d) in three patients. Breast tissue in categories c and d was defined as dense, whereas that in the other categories was defined as non-dense.

**Fig. 6:**
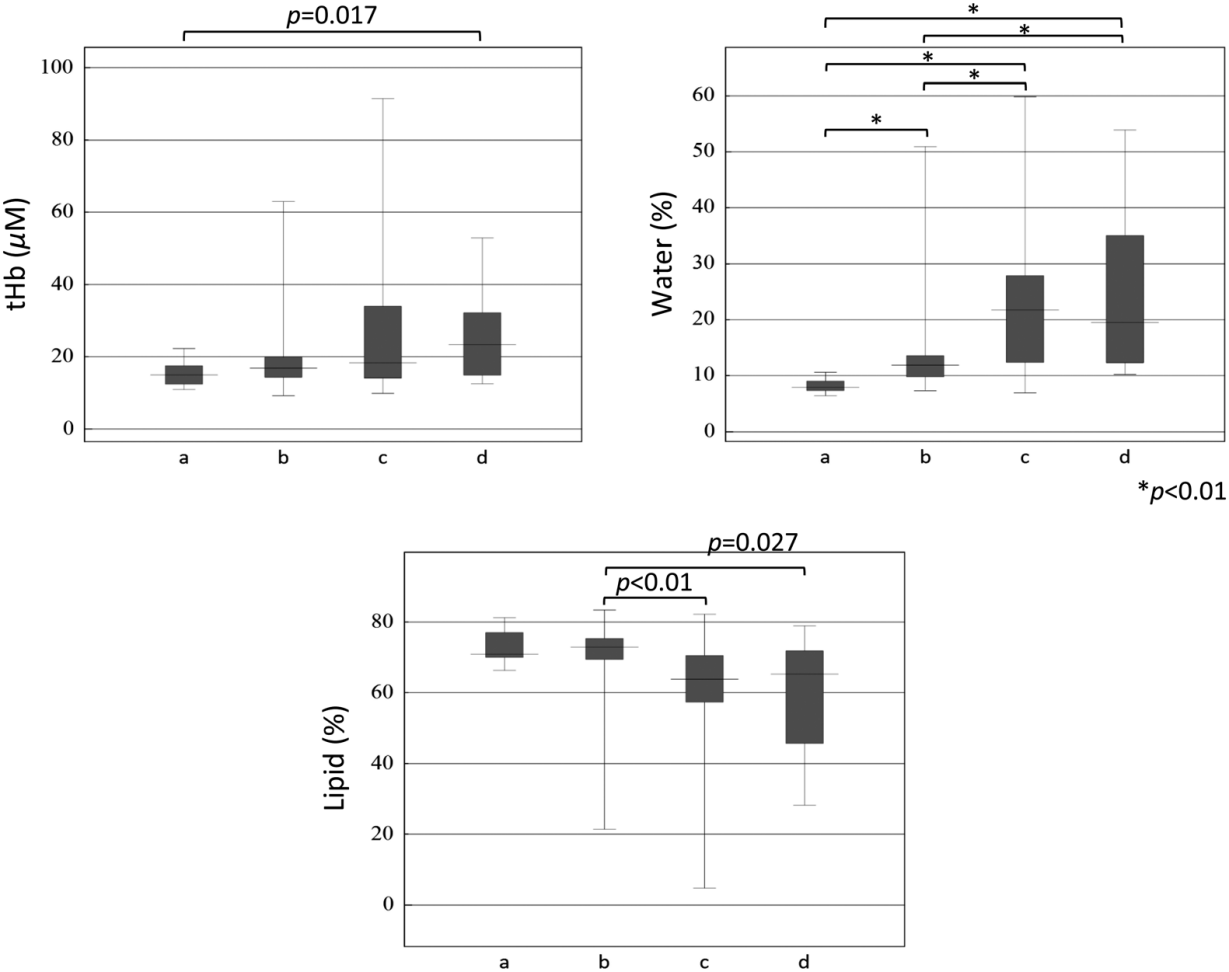
Boxplots of tHb concentration, water content, and lipid content for each mammography category. The median value of tHb was 14.9  μM in category a (almost entirely fat), 16.8  μM in category b (scattered fibroglandular densities), 18.3  μM in category c (heterogeneously dense), and 23.3μM in category d (extremely dense). The tHb concentration in category a was significantly lower than that in d (p=0.017). The median water content was 7.9% in category a, 11.9% in category b, 21.8% in category c, and 19.5% in category d. Although there was no significant difference between the water content in categories c and d, significant differences were found for all other pairwise group comparisons (p<0.01). The median values of lipid content were 70.9% in category a, 72.9% in category b, 63.8% in category c, and 65.3% in category d. The lipid content in category b was significantly higher than that in category c (p<0.01) and category d (p=0.027).

**Fig. 7:**
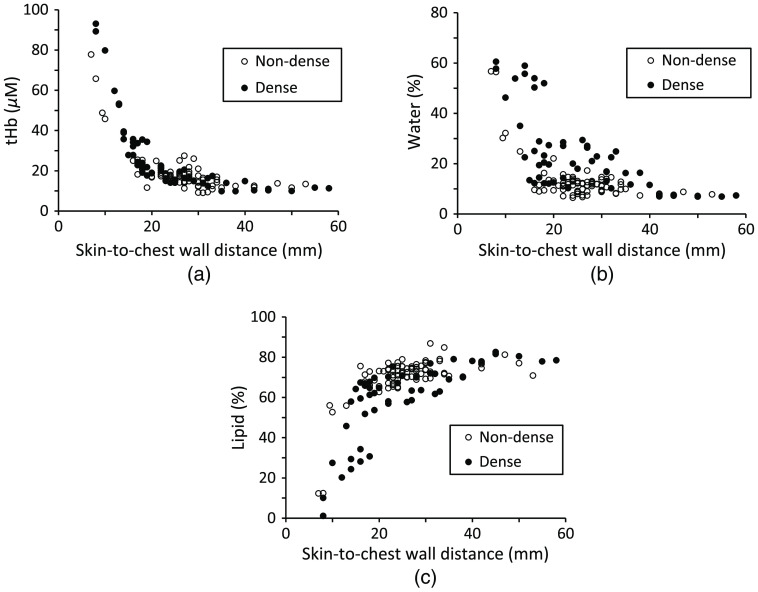
(a) tHb concentration, (b) water content, and (c) lipid content versus skin-to-chest wall distance in normal breast tissue for non-dense (n=83) and dense (n=50) breasts.

Boxplots of the tHb concentration, water content, and lipid content in normal breast tissue for each category are shown in Fig. 6. There were 14 data in category a, 69 in category b, 36 in category c, and 14 in category d. The tHb concentration in category a was significantly lower than that in category d (p=0.017). Although there was no significant difference between the water content in categories c and d, significant differences in water content were found for all other pairwise group comparisons (p<0.01). The lipid content in category b was significantly higher than that in category c (p<0.01) and category d (p=0.027). Figure 7 shows the relationship between the skin-to-chest wall distance and tHb concentration, water content, and lipid content of normal breast tissue in non-dense (n=83) and dense breasts (n=50). As the skin-to-chest wall distance decreased, the tHb concentration and water content increased for both non-dense and dense breasts. There was a tendency for the water content to be higher in dense breasts than in non-dense breasts for a given skin-to-chest wall distance. The lipid content decreased as the skin-to-chest wall distance decreased. Moreover, there was a tendency for the lipid content to be lower in dense breasts than in non-dense breasts for a given skin-to-chest wall distance. The skin-to-mammary gland distance was shorter in dense breasts than in non-dense breasts (p<0.001), with a median distance of 10 mm [interquartile range (IQR): 7–14, n=50] in dense breasts and 14 mm (IQR: 11–18, n=83) in non-dense breasts.

**Fig. 8:**
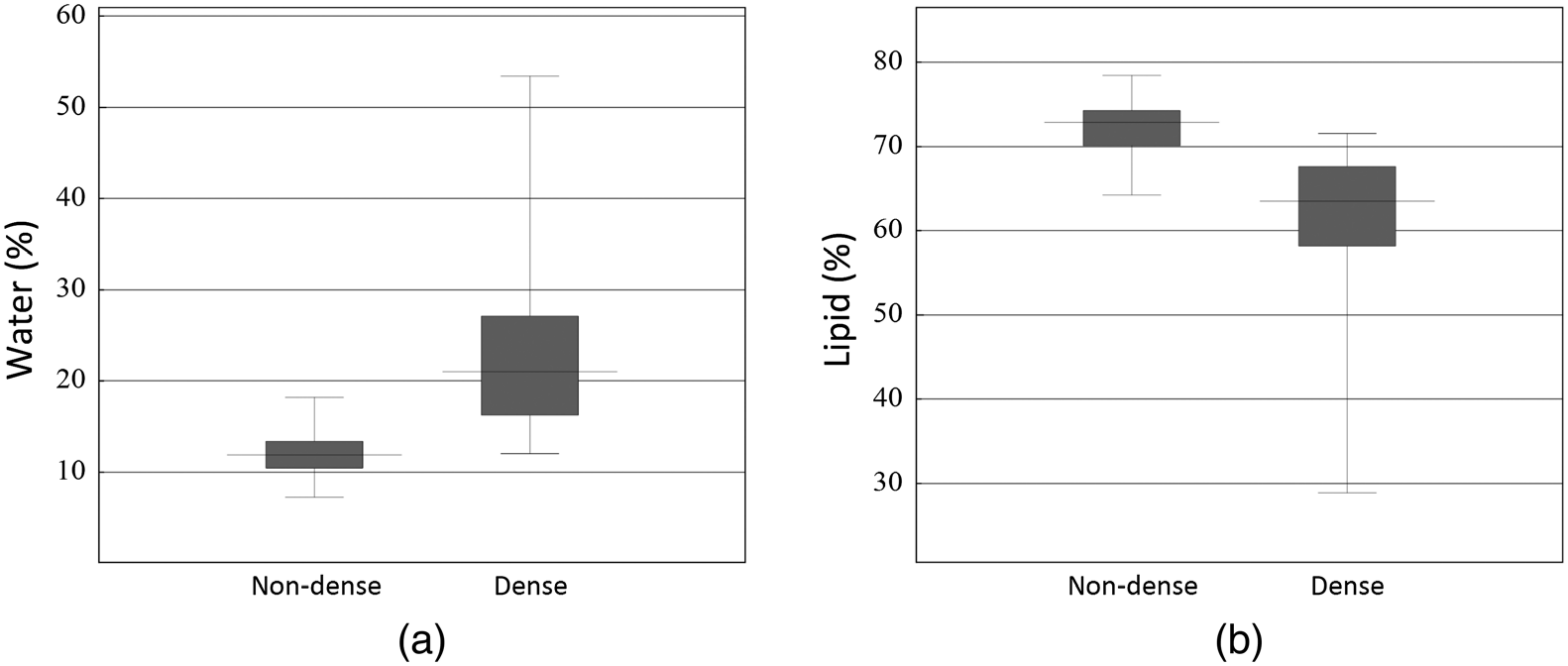
Boxplots of (a) water content and (b) lipid content of non-dense (n=52) and dense (n=31) breasts with a skin-to-chest wall distance of 15 mm or more and a skin-to-mammary gland distance of 15 mm or less, assuming that these data reflect the content of breast tissue with a mammary gland and are minimally influenced by the chest wall. The water content of normal breast tissue was higher in dense breasts than in non-dense breasts (p<0.001). The lipid content of normal breast tissue was lower in dense breasts than in non-dense breasts (p<0.001).

According to Fig. 3, the depth sensitivity for water and lipid content was ∼15  mm. Figure 8 shows boxplots of water and lipid content for non-dense (n=52) and dense (n=31) breasts with a skin-to-chest wall distance of 15 mm or more and a skin-to-mammary gland distance of 15 mm or less, assuming that these data reflect the content of breast tissue with a mammary gland and are minimally influenced by the chest wall. The water content in normal breast tissue was higher in dense than in non-dense breasts (p<0.001), with a median content of 21.0% (IQR: 16.2–27.1, n=31) in dense breasts and 11.9% (IQR: 10.5–13.4, n=52) in non-dense breasts. The lipid content in normal breast tissue was lower in dense breasts than in non-dense breasts (p<0.001), with a median content of 63.5% (IQR: 58.2–67.6, n=31) in dense breasts and 72.9% (IQR: 70.1–74.3, n=52) in non-dense breasts.

### 3.5 ROC Analysis for Distinguishing Dense from Non-Dense Breasts

**Fig. 9:**
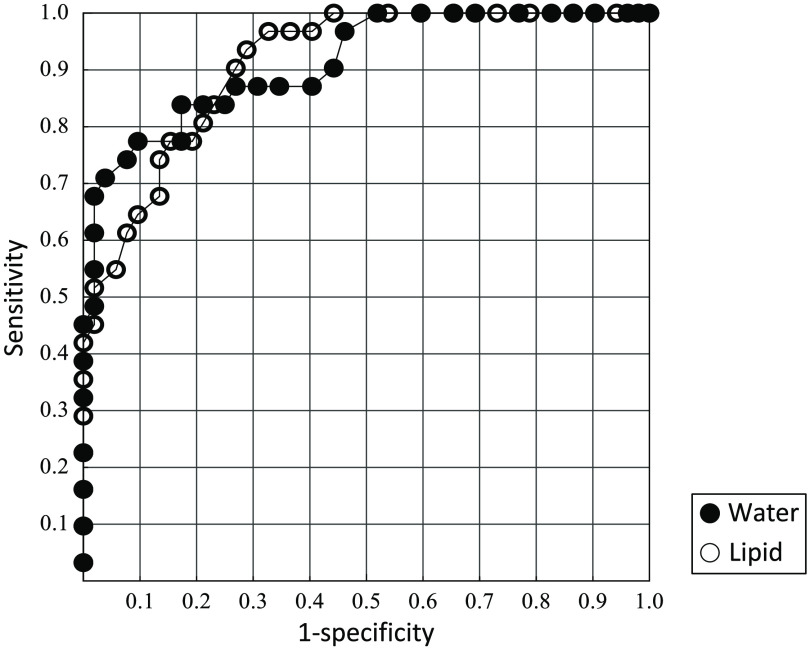
ROC curves for 83 data corresponding to a skin-to-chest wall distance of 15 mm or more and a skin-to-mammary gland distance of 15 mm or less. The AUC was 0.91 for water content and 0.91 for lipid content.

ROC curve analysis was used to evaluate the possibility of distinguishing dense from non-dense breasts based on optical parameters. Figure 9 shows the ROC curve for normal breast tissue for 83 data (31 dense and 52 non-dense breasts) with a skin-to-chest wall distance of 15 mm or more and a skin-to-mammary gland distance of 15 mm or less, assuming that these data reflect the content of breast tissue with a mammary gland and are minimally influenced by the chest wall. The AUC was 0.91 for both water content and lipid content. With an optimal cutoff of 14.0% for water content, the results were: diagnostic accuracy, 83.1%; sensitivity, 83.9%; specificity, 82.7%; PPV, 74.3%; and NPV, 89.6%. With an optimal cutoff of 69.1% for lipid content, the results were: diagnostic accuracy, 80.7 %; sensitivity, 80.6%; specificity, 80.8%; PPV, 71.4%; and NPV, 87.5%.

## 4 Discussion

In previous studies, we conducted hemoglobin measurements in breast cancer patients using a three-wavelength TD-DOS system and reported the influence of the chest wall muscles.^9^^,^^10^ In this study, we performed measurements with a six-wavelength TD-DOS system to evaluate water and lipid content.

As shown in Fig. 3, the tHb concentration and water content increased and lipid content decreased as the skin-to-chest wall distance decreased for a two-layer structure of a lipid layer and chest wall. The results suggest that the chest wall contains high amounts of tHb and water, with a lower lipid content. A high concentration of tHb in the chest wall could be caused by myoglobin, which is measured as the hemoglobin concentration with the TD-DOS system.^9^ The results also show that the lipid layer contains some water and hemoglobin. In the evaluation of tHb concentration, water content, and lipid content in the two-layer structure of a lipid layer and chest wall, the depth sensitivity of the TRS-21-6W system with a source–detector separation of 3 cm was revealed based on the relationships between the parameters and the skin-to-chest wall distance. Figure 3 shows that the water and lipid content reached a plateau at a skin-to-chest wall distance of ∼15  mm, which is slightly smaller than the value obtained for tHb (∼20  mm).

As shown in Fig. 4, the results for all 133 data showed the same trends for the tHb concentration, water content, and lipid content seen for the two-layer structure. From the fitted curves, the R value for the tHb concentration was higher than that for the water content. The coefficient of variation also indicated that the water content had a larger variation than tHb concentration when the skin-to-chest wall distance exceeded 20 mm (i.e., for distances at which the chest wall is thought to have little effect on both water content and tHb concentration). The R value for tHb concentration in two-layer structure and normal breast (three-layer structure with fat, mammary gland, and chest wall) were 0.978 and 0.938, respectively. Similarly, the R values were 0.965 and 0.774 for water content and 0.931 and 0.838 for lipid content. Because the R values for water and lipid content decreased in the three-layer structure, it would be suggested that the mammary gland had a large influence on water and lipid content. The mammary gland, which lies above the chest wall, should cause larger individual differences in the water and lipid content than in the tHb concentration.

In contrast, as shown in Fig. 5, the R value for the tHb concentration was smaller than that for water content. The tHb values showed greater variation than the water content when the skin-to-mammary gland distance was smaller than 10 mm (Fig. 5). This result suggests that the influence of the chest wall on the measured tHb varies. The distribution of water and lipid content against the skin-to-mammary gland distance resembles that of a two-layer structure, suggesting that the influence of the chest wall on the measured water and lipid content is small.

In Fig. 6, the tHb concentrations in each MMG category were significantly different only between the fatty and extremely dense groups. The water content was significantly different between most MMG categories, and the lipid content was significantly different between some MMG categories of breast density. In Fig. 7, the tHb concentration was plotted against the skin-to-chest wall distance in dense and non-dense breasts separately, with the value in dense breasts looks slightly higher than that in non-dense breasts. The chest wall appears to have a strong influence on tHb, but there was a small difference between the tHb concentrations in non-dense and dense breasts. In contrast, there were differences between non-dense and dense breasts for the water or lipid content. The water content was higher for dense than non-dense breasts, whereas the lipid content was lower in dense than in non-dense breasts when the content of breast tissue reflecting mammary gland and with little effect from the chest wall.

Finally, we analyzed the ROC curve for water and lipid content to evaluate the potential of the TRS-21-6W system to discriminate dense from non-dense breasts. Because the depth sensitivity of the system with a source–detector separation of 3 cm was ~15 mm for the water and lipid content of the breast, it is assumed that the result reflected only the influence of the lipid layer when an area far from the mammary gland was measured. With this assumption, we analyzed the data corresponding to a skin-to-chest wall distance of 15 mm or more and a skin-to-mammary gland distance of 15 mm or less, which reflect the water and lipid content of the mammary gland with little effect from the chest wall. The result shows that dense and non-dense breasts could be distinguished with high sensitivity and specificity based on their water and lipid content. There was a tendency for the skin-to-mammary gland distance to be smaller in dense breasts than in non-dense breasts, and this difference may have influenced the results of the ROC analysis. The device showed the potential to distinguish dense from non-dense breasts.

The depth sensitivity for measuring water and lipid content was approximately 15 mm with a source–detector separation of 3 cm, which is important when measuring these values in breast cancers using the TRS-21-6W system.

There is an issue to address with this study: data for skin-to-mammary gland distances close to 15 mm are thought to almost only reflect the influence of the lipid layer. The distribution of the mammary gland is uneven and sometimes asymmetrical.^31^ The assessment of data at distances far enough to have a weaker effect from the chest wall and distances small enough to capture the effect of the mammary gland might result in a better understanding of the mammary gland structure. It is necessary to assess the appropriate assumptions for measuring normal breast tissue.

To measure the water and lipid content with improved depth sensitivity, the source–detector separation should be increased. We are currently evaluating data obtained using different source–detector separations.

## 5 Conclusion

In this study, we performed measurements using a six-wavelength TD-DOS system and evaluated the water and lipid content in conjunction with the structural information of breast tissue provided by US. We also demonstrated the possibility of distinguishing dense from non-dense breasts by reflectance measurements with this six-wavelength TD-DOS system. Since the water and lipid content had smaller influence of the chest wall than the tHb concentration, the system should be suitable for evaluating mammary glands.
